# A DFT-Based Quantitative and Geometric Analysis of the Effect of Pressure on Boron Arsenate

**DOI:** 10.3390/ma15144858

**Published:** 2022-07-12

**Authors:** James N. Grima-Cornish, Liana Vella-Żarb, Joseph N. Grima, Kenneth E. Evans

**Affiliations:** 1 Metamaterials Unit, Faculty of Science, University of Malta, MSD 2080 Msida, Malta; joseph.grima@um.edu.mt; 2 Department of Chemistry, Faculty of Science, University of Malta, MSD 2080 Msida, Malta; liana.vella-zarb@um.edu.mt; 3 College of Engineering, Mathematics and Physical Sciences, University of Exeter, Exeter EX4 4QF, UK; k.e.evans@ex.ac.uk

**Keywords:** negative linear compressibility, auxetic, mechanical metamaterials

## Abstract

Boron arsenate, BAsO_4_, is a β-cristobalite-like crystal which has been reported to exhibit the rather unusual property of negative linear compressibility behaviour at elevated pressures, that is expanding rather than shrinking in a linear dimension when subjected to pressure. This work proposes a ‘geometry—deformation mechanism’-based mathematical model to aid the discernment of the manner how this anomalous pressure behaviour is achieved. The model makes use of data obtained from DFT simulations over an extended range of pressures, including extreme pressure conditions, and rigorously explains the macroscopic properties of this material in terms of the nanoscale deformations. More specifically, through this model, it was possible to decipher the different contributions to the deformation mechanism and compressibility properties of BAsO_4_. Moreover, for the first time, it was shown that a rule related to the sum of angles of tetrahedrally coordinated atoms is so robust that it applies at the extreme pressures studied here.

## 1. Introduction

Boron arsenate, BAsO_4_, is a β-cristobalite-like crystal, with an $I\bar{4}$ symmetry, and is composed of boron tetroxide (BO_4_) and arsenic tetroxide (AsO_4_) tetrahedra [1,2]. In recent years, this material has been extensively studied from the perspective of its mechanical behaviour and found to exhibit various anomalous properties [3,4,5,6]. In particular, almost two decades ago, Haines et al. [3] were able to identify for the first time the anomalous response of linear expansion rather than contraction in the *c* crystallographic direction when the material is subjected to an increase in pressure, a property which was later termed ‘negative linear compressibility’ (NLC). This anomalous compressibility property was measured through a combination of experimental work and theoretical density functional theory (DFT)-based studies and is exhibited at moderately high non-ambient pressures of *c*. This study also reported a large shrinkage in the *a* and *b* crystallographic directions which was attributed to tetrahedral rotations.

A more recent study by Grima-Cornish et al. [4] re-attributed the NLC in the *c* crystallographic direction to deformations of the BO_4_ and AsO_4_ tetrahedra which tend to become more elongated when subjected to an increase in pressure rather than the more prominent tetrahedral rotations [4]. This mechanism leading to NLC is somewhat reminiscent of the well-known ‘wine-rack’ mechanism [7] which adequately explains the manifestation of NLC in a number of molecular-level systems. However, it is noticeably distinct, as the wine-rack motif is not actually present. Instead, NLC results merely from the closure of V-like atomic units, i.e., similar to a triangular elongation, an effect which can be considered as a half wine-rack, and was hence termed a ‘demi wine-rack’ mechanism. In the same work, it was shown that these pronounced tetrahedral rotations, which almost overshadow the demi-wine rack mechanism, contribute to another anomalous property: auxeticity [8]. This is the phenomenon of expanding laterally when uniaxially stretched (negative Poisson’s ratio, NPR). In fact, as explained by Grima-Cornish et al. [4], simulations suggest that the auxetic behaviour which is primarily present in the (001) plane can be explained in terms of a ‘rotating squares’ model [9,10] which represents the two-dimensional projection of the three-dimensional tetrahedra which also rotate [11,12,13]. Properties such as negative Poisson’s ratios and/or negative linear compressibility are known to be important in various practical applications, and hence have been the subject of extensive research in recent years as discussed elsewhere [10,14,15,16,17,18]. Some of these studies, particularly ones on negative Poisson’s ratios, examine auxetics through model structures and macromodels [8,9,10,19,20,21,22,23,24,25,26,27,28,29,30,31,32,33,34,35,36,37,38,39,40,41,42,43,44,45], an approach which was found to be highly useful in elucidating the properties of such unusual systems. Significant advances on the subject of negative compressibility have also been made [7,17,46,47,48,49,50,51,52,53], as well as on potential applications (e.g., smart textiles [54] biomedical devices [55,56], etc.).

Despite these important findings on boron arsenate, there are still various features which have not yet been investigated or explained. For example, the existing publications lack a detailed quantitative interpretation from a mechanistic and geometric perspective of the data that was obtained from experiment and simulation, partly due to the fact that the main scope of earlier work was to report the phenomena of negative mechanical properties. Furthermore, in all studies so far, the range of pressures applied were capped at 60 GPa with the result that the behaviour of this material at extreme pressures is still unknown.

This work aims to address these issues through the analysis of simulated crystallographic data generated using the Cambridge Sequential Total Energy Package (CASTEP) [57,58] Density Function Theory (DFT) module within the Materials Studio (BIOVIA, Dassault Systèmes, San Diego, CA, USA). These data, which represent a much extended dataset of what was reported earlier using the same protocol [4], are analysed via a newly derived mathematical model which describes BAsO_4_ parametrically thus permitting a better interpretation of the existing and newly reported data including elucidation of the causes of NLC, or lack thereof. This gives a quantitative insight into some still untacked phenomena at moderate and extreme pressures.

## 2. Boron Arsenate at Ambient Pressures

### 2.1. An Analysis of BAsO*_4_* in Terms of Its Geometric Features

#### 2.1.1. General Considerations

**Figure 1:**
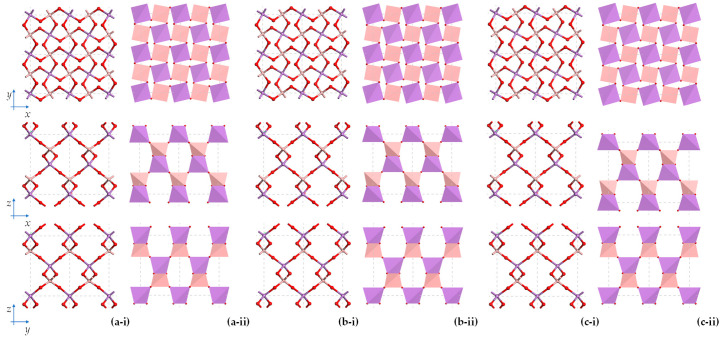
Images of the structure of BAsO_4_ at *p* = 1 Atm = 0.101 GPa depicted in terms of (**i**) atoms (spheres) and bonds (cylinders), and (**ii**) tetrahedra comparing the experimentally obtained crystal structure of BAsO_4_ as obtained by (**a**) Shulze [2] and (**b**) Haines et al. [3] with that simulated using the DFT simulations, (**c**). Details of the protocol used for the simulations are outlined in Appendix A.

Figure 1 illustrates the crystal structure of BAsO_4_ as determined experimentally by Schulze et al. [1,2] and Haines et al. [3], alongside the crystal structure at ambient pressure as simulated in this study through the DFT simulations carried out using the CASTEP module within Materials Studio. Note that this figure clearly shows that the simulation protocol as applied in this work (see Appendix A) replicates perfectly the crystal structure of this material at ambient pressure conditions.

As illustrated in Figure 1, BAsO_4_ comprises of two types of corner-sharing tetrahedra which at ambient pressures project in the (001) with the characteristic ‘rotating squares’ geometry. More specifically, a 1 × 1 × 1 unit cell of the BAsO_4_ crystal contains two BO_4_ and two AsO_4_ tetrahedra where, at ambient pressures, the two smaller BO_4_ tetrahedra are equal to each other, and the two larger AsO_4_ tetrahedra are also equal to each other, as expected from the $I\bar{4}$ symmetry. Before proceeding with the analysis, a distinction should be made between the parameters that define and describe the shape of the tetrahedra, and parameters which relate to their relative orientation in space. In particular, the intra-tetrahedral bond angles, which relate to a single tetrahedron (the O-As-O and O-B-O ‘intra-tetrahedral angles’) alongside the bond lengths As-O and B-O, define the tetrahedral shapes. On the other hand, bond angles between two adjacent tetrahedra (the As-O-B ‘inter-tetrahedral angles’) do not affect the shape of the tetrahedra, but only their orientation. Moreover, the shape and size of these four tetrahedra within the unit cell can be completely described in terms of their respective O-As and O-B bond lengths and O-As-O and O-B-O bond angles, which can in turn be used to compute the length of the six edges (O-O) and the twelve angles at the vertices (O-O-O) for each tetrahedron.

#### 2.1.2. On the Shape of Tetrahedra under Ambient Pressures

Although a cursory look may indicate that the tetrahedra within the structure are regular, this is in fact not the case. In fact, although for BAsO_4_ under ambient pressure the bond lengths within each tetrahedron are equal to each other, the vertex-centre-vertex bond angles O-As-O and O-B-O are not equal to arccos(−1/3) ≈ 109.47°, the O-O-O vertex angles are not 60°, the edges are not of equal length and the faces are not all equal (i.e., congruent) equilateral triangles.

Instead, the simulated data in this study as well as the experimental data by Haines et al. [3] and earlier data by Schulze et al. [1,2] suggest that for all tetrahedra in the system, two non-touching vertex-centre-vertex bond angles (henceforth referred to as the angles in ‘Set 1’) are equal but larger than the other four angles (henceforth referred to as the angles in ‘Set 2’) with the consequence that at ambient pressures the two non-touching edges of the tetrahedron opposite to these larger angles (henceforth referred to as ‘Set 1’ edges) are equal but longer than the other four edges (henceforth referred to as ‘Set 2’ edges). Such tetrahedra are classifiable as ‘tetragonal disphenoids’ i.e., tetrahedra which, by definition, have four equal faces in the shape of isosceles triangles [59] and exhibit D_2d_ dihedral symmetry.

Tetragonal disphenoids are characterised with some rather interesting geometric features, including the ability to project as a square in two dimensions. In such a projection, the four equal sides would project as the sides of the square while the remaining two sides would project as the diagonals. The plane of this projected square lies parallel to the aforementioned two equal sides of the tetrahedron which correspond to the diagonals of the square. This is very evident from Figure 1.

**Figure 2:**
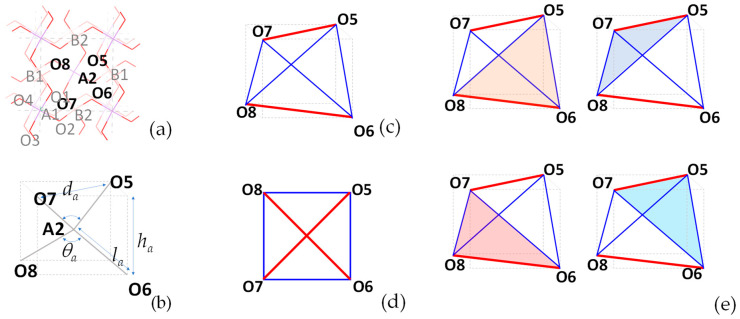
(**a**) The unit cell of BAsO_4_ with the atoms labelled, (**b**) an illustration of how this tetrahedron, which is in the shape of a tetragonal disphenoid, is representable as atoms and bonds, and how it fits within a cuboid with a square upper and lower face; (**c**) a representation of this tetrahedron in terms of the edges where the two equal Set 1 edges are shown in red and the four equal Set 2 edges are shown in blue, (**d**) the 2D projection of this tetrahedron, looking from above, to highlight the 2D projection of a square (which in BAsO_4_ corresponds to the (001) plane); and (**e**) a highlight of the four congruent triangular faces of the tetrahedron.

To explain these geometric relationships, without loss of generality, one may look at the tetrahedron (tetragonal disphenoid) at the centre of the unit cell, i.e., the AsO_4_ tetrahedron having A2 at its centre and O5, O6, O7 and O8 as its vertices (see Figure 2). In this case, the two equal longer sides are O5-O7 and O6-O8, corresponding to the larger bond angles O5-A2-O7 and O6-A2-O8 (Set 1) whilst the other four equal shorter sides are O5-O6, O6-O7, O7-O8 and O8-O5, corresponding to the smaller bond angles O5-A2-O6, O6-A2-O7, O7-A2-O8 and O8-A2-O5 (Set 2). This tetrahedron has four faces, each of which is an isosceles triangle, all of which are congruent. Moreover, as illustrated in Figure 2c, this tetrahedron may be fitted into a cuboid with a square base, where atoms O5 and O7 occupy opposite corners of the top square face whilst the atoms O6 and O8 occupy the other opposite corners on the bottom square face with the result that, when viewed from above, this tetrahedron projects as a square in the plane parallel to the edges O5-O7 and O6-O8. These edges O5-O7 and O6-O8 correspond to the diagonals of the projected square (shown in red in Figure 2), whilst the ‘shadows’ of the other edges correspond to the sides of the projected square (shown in blue in Figure 2). Note that such geometric features are shared by all tetragonal disphenoids, including the three other tetrahedra within the unit cell. Note also that similar properties would also be manifested had the angles O5-A2-O7 and O6-A2-O8 in Set 1 been smaller than the other four angles in Set 2 (which is not the case at ambient pressure), with the ‘diagonal’ edges O5-O7 and O6-O8 always corresponding in full to the diagonals of the projected squares whilst the ‘shadows’ of O5-O6, O6-O7, O7-O8 and O8-O5 (which have a shorter length than the edges themselves) correspond to the sides of the squares. Note also that as a consequence of these features, provided that the tetrahedron remains a tetragonal disphenoid, the edges O5-O7 and O6-O8 necessarily point in two orthogonal directions and form the diagonals of the projected square. Moreover, the line joining the mid-points of these two edges is perpendicular to the two edges with the A2 arsenic atom lying in its centre (a position which corresponds to the location of the centre of the tetrahedron). In other words, the central atom within the tetrahedron bisects a line which is the perpendicular bisector of the two Set 1 edges. Another interesting phenomenon that should be noted is that for each tetrahedron in our system, the sum of the six O-As-O or O-B-O bond angles sum up to approximately 657° (i.e., ≈6 × 109.47°), which as noted by McNelis and Blandino [60] from their examination of many tetrahedral systems, seems to be a value which applies to practically all tetrahedral molecular units.

#### 2.1.3. On the Relative Orientation of Tetrahedra under Ambient Pressures

Apart from analysing the shape and size of the tetrahedra, it is also important to examine the relative orientation of the tetrahedra in space. The first feature which should be highlighted is that at ambient pressures, the four tetragonal disphenoids in the unit cell are oriented in such a way that the two ‘Set 1’ longer edges which correspond to the diagonals of the projected squares all lie parallel to the (001) crystallographic plane. This means that the two larger vertex-centre-vertex bond angles always lie in planes perpendicular to the (001) plane. As a result, the tetrahedral framework projects with the familiar ‘connected squares’ motif in this (001) plane where the squares are of two different sizes [9], the larger ones corresponding to the projections from the AsO_4_ tetrahedra and the smaller ones to the BO_4_ tetrahedra.

**Figure 3:**
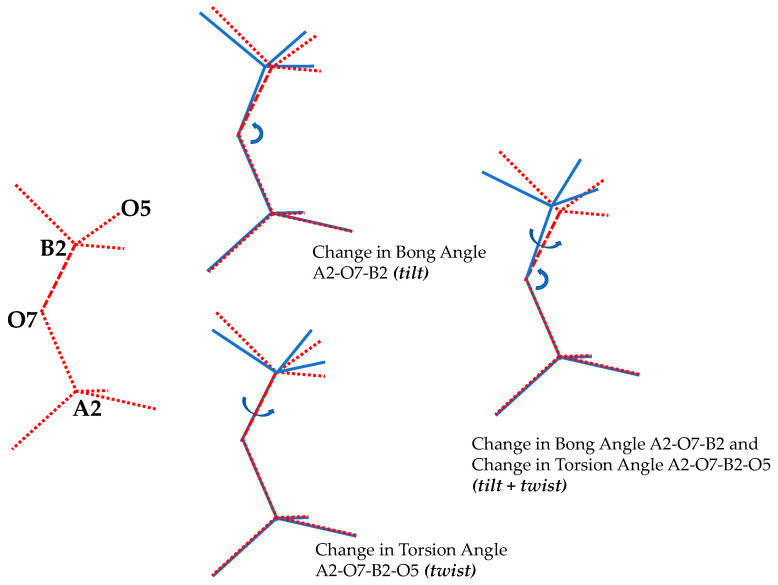
The effect of a change in As-O-B bond angle tetrahedral rotation as a ‘tilt’ compared to a change in ‘twist’ measured through a change in torsion angle tilt.

Moreover, it should be noted that the relative orientation of the tetrahedra in space may be discussed in terms of the inter-tetrahedral As-O-B bond angles and the torsion angles, which must always involve two connected tetrahedra. In simple terms, as shown schematically in Figure 3, a change in the As-O-B bond angles would constitute a ‘tilt’ of the tetrahedra whilst a change in the torsion angles constitute a ‘twist’ around the axis defined by the middle bond. In this particular case, an interesting observation that should be made is that at ambient pressure conditions, all As-O-B angles are equal to each other, as are the torsion angles. What should also be noted is that any two directly connected tetrahedra would not occupy the same level of depth down the [001] crystallographic axis (which corresponds to the global *z*-axis). In fact, from the experimental data, going up the *z*-direction from (*x*,*y*,*z*) = (0,0,0) one first encounters the A1-centred tetrahedra (A1 atom lying at *z* = 0), then the B1-centred tetrahedra (B1 atom lies at *z* = ¼ *c*), then the A2-centred tetrahedra (A2 atom lies at the centre of the unit cell, i.e., at *z* = ½ *c*), then the B2-centred tetrahedra (the B2 atom lies at *z* = ¾ *c*) and finally again the A1-centred tetrahedra in the top corners (*z* = *c*).

### 2.2. Mathematical Expressions for the Cell Parameters of BAsO*_4_* at Ambient Pressures

#### 2.2.1. Derivation

Given the manner of how the tetrahedra are oriented in space and their relative stacking, at ambient conditions, the c dimension should be computable from the sum of the height *h* of the four tetrahedra (*h* = *h_a_* for the specific tetrahedron shown in Figure 2). For tetragonal disphenoids, as shown in Figure 2, this height h, measured as the shortest distance between the Set 1 edges may be related to the bond length l and the bond angle *θ* in Set 1 (where *l* = *l_a_* and *θ* = *θ_a_* for the specific tetrahedron shown in Figure 2) by:

(1) $$h=2l\cos\left(\frac{\theta }{2}\right),$$

Thus, according to this prediction, since the two AsO_4_ tetrahedra are identical to each other and the two BO_4_ are also identical to each other, then the *c* parameter should be estimable from the heights of the tetrahedra, as follows:

(2) $$c_{eqn}=2h_{a}+2h_{b},$$

where *h_a_* and *h_b_* are the respective heights of the AsO_4_ and BO_4_ tetrahedra, which may be expressed in terms of *l_a_* and *l_b_* (the As-O and B-O bond lengths respectively) and *θ_a_* and *θ_b_* (the O-As-O and O-B-O Set 1 bond angles respectively) as follows:

(3) $$h_{a}=2l_{a}\cos\left(\frac{\theta_{a}}{2}\right),$$

and:

(4) $$h_{b}=2l_{b}\cos\left(\frac{\theta_{b}}{2}\right),$$

i.e.,:

(5) $$c_{eqn}=4l_{a}\cos\left(\frac{\theta_{a}}{2}\right)+4l_{b}\cos\left(\frac{\theta_{b}}{2}\right),$$

**Figure 4:**
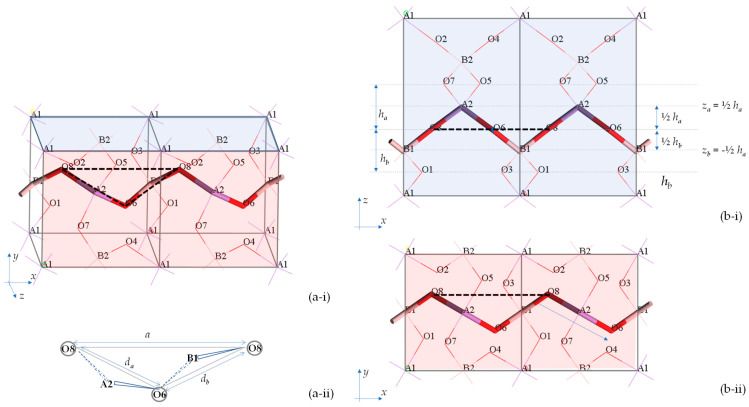
The method by which the *a*-direction was computed from bond lengths and bond angles. (**a**) Depicts the crystal in three dimensions, where (**a-i**) shows 2 × 1 × 1 units cells and (**a-ii**) shows in more detail the relationship between the parameter a and the parameters *d_a_* and *d_b_*; whereas (**b**) shows the same system as shown in the (**b-i**) *zx-*plane and (**b-ii**) *xy*-plane.

Having established these basic facts on the manner how the structure of BAsO_4_ is composed, in terms of the shape of the tetrahedra and the way they connect and stack down the third direction, one may discern more elegantly what gives this crystalline material its characteristic ‘rotating squares’ motif when viewed down the [001] direction. First are foremost, as explained above, and evident from a comparison of Figure 1 and Figure 2, each ‘projected’ square corresponds to an individual tetrahedron and the square shape of the projection is a direct geometric consequence of having the tetrahedra in the shape of tetragonal disphenoids. Moreover, two vertex sharing tetrahedra will each have a Set 1 edge which is co-planar to each other. As shown in Figure 4, such co-planar edges span the length of the unit cell from one face to the other, i.e., one can calculate the *a* and *b* unit cell lengths from knowledge of the length of these tetrahedral edges (which correspond to the diagonals of the 2D squares) and the angle between them, in which the angle is the 2D projection in the (001) plane of a B-O-As bond angle.

Applying the triangle cosine rule for the triangle which forms from two such diagonals and a unit-cell edge, one obtains:

(6) $$a_{eqn}=b_{eqn}=\sqrt{d_{a}^{2}+d_{b}^{2}-2d_{a}^{}d_{b}^{}\cos\left(\theta_{2d}\right)},$$

where *a_eqn_* and *b_eqn_* are the unit cell lengths *a* and *b* calculated in this manner, *d_a_* and *d_b_* are the lengths of the Set 1 edges of the AsO_4_ and BO_4_ tetrahedra respectively and *θ*_2*d*_ is the angle between these edges (diagonals), which is the 2D projection in the (001) plane of the B-O-As bond of magnitude *θ*_3*d*_. The terms *d_a_* and *d_b_* may be expressed in terms of bond lengths and bond angles as follows:

(7) $$d_{a}=2l_{a}\sin\left(\frac{\theta_{a}}{2}\right),$$

(8) $$d_{b}=2l_{b}\sin\left(\frac{\theta_{b}}{2}\right),$$

whilst, as shown below, the 2D projected angle *θ*_2*d*_ may be related to the As-O-B angle *θ*_3*d*_ and the O-As-O and O-B-O bond angles, *θ_a_* and *θ_b_*.

Without loss of generality, we derive *θ*_2*d*_ for the specific case of the angle between the edge O8-O6 in the AsO_4_ tetrahedron with centre atom A2 and edge O6-O8 in the BO_4_ tetrahedron with centre atom B1, and in the process we obtain an expression for the cell parameters *a* and *b* in terms of the bond lengths *l_a_* and *l_b_*, and the bond angles *θ_a_*, *θ_b_* and *θ_3d_*.

By noting that the bonds A2-O6 and O6-B1 may be represented in vector form as:

(9) $$\vec{A2\text{-}O6}=\left(\begin{array}{c} x_{a}\\ y_{a}\\ z_{a} \end{array}\right),\;\vec{O6\text{-}B1}=\left(\begin{array}{c} x_{b}\\ y_{b}\\ z_{b} \end{array}\right)$$

where:

(10) $$\left|\vec{A2\text{-}O6}\right|=l_{a},\;\left|\vec{O6\text{-}B1}\right|=l_{b}$$

i.e.,

(11) $$\left(\vec{A2\text{-}O6}\right).\left(\vec{O6\text{-}B1}\right)=x_{a}x_{b}+y_{a}y_{b}+z_{a}z_{b}=l_{a}l_{b}\cos\left(\theta_{3d}\right)$$

and by further noting that in this special case where the tetrahedra are tetragonal disphenoids and the atoms O8 and O6 have the same z-coordinate, i.e., one may write diagonals O8-O6 (relating to the tetrahedron with centre atom A2) and O6-O8 (relating to the tetrahedron with centre atom B1) in vector form, respectively, as:

(12) $$\vec{O8\text{-}O6}=\left(\begin{array}{c} 2x_{a}\\ 2y_{a}\\ 0 \end{array}\right),\;\vec{O6\text{-}O8}=\left(\begin{array}{c} 2x_{b}\\ 2y_{b}\\ 0 \end{array}\right),$$

where $\left|\vec{O8\text{-}O6}\right|=d_{a}$ and $\left|\vec{O6\text{-}O8}\right|=d_{b}$, i.e.,:

(13) $$\left(\vec{O8\text{-}O6}\right).\left(\vec{O6\text{-}O8}\right)=4x_{a}x_{b}+4y_{a}y_{b}=d_{a}d_{b}\cos\left(\theta_{2d}\right).$$

Thus, from Equations (11)–(13) we obtain:

(14) $$d_{a}d_{b}\cos\left(\theta_{2d}\right)-4l_{a}l_{b}\cos\left(\theta_{3d}\right)=\left(4x_{a}x_{b}+4y_{a}y_{b}\right)-4\left(x_{a}x_{b}+y_{a}y_{b}+z_{a}z_{b}\right),$$

i.e.,

(15) $$d_{a}d_{b}\cos\left(\theta_{2d}\right)=4l_{a}l_{b}\cos\left(\theta_{3d}\right)-4z_{a}z_{b}.$$

But from geometric considerations for a system where the tetrahedra are tetragonal disphenoids and the specfic manner of how they are connected, i.e., in a manner where As and B atoms are located at the midpoint of the perpendicular bisector of the edges of the two connceted tetrahedra, one pointing up in the *z*-direction, one pointing down, the term $4z_{a}z_{b}$ may be re-written in terms of the heights of the tetrahedra as:

(16) $$4z_{a}z_{b}=-h_{a}h_{b},$$

i.e.,

(17) $$d_{a}^{}d_{b}^{}\cos\left(\theta_{2d}\right)=4l_{a}^{}l_{b}^{}\cos\left(\theta_{3d}\right)+h_{a}^{}h_{b}^{},$$

i.e., from (15) substituted into (6) we obtain:

(18) $$a_{eqn}=b_{eqn}=\sqrt{d_{a}^{2}+d_{b}^{2}-8l_{a}^{}l_{b}^{}\cos\left(\theta_{3d}\right)-2h_{a}^{}h_{b}^{}},$$

i.e., by further substituting (3), (4), (7) and (8) into (18):

(19) $$a_{eqn}=b_{eqn}=\sqrt{4l_{a}^{2}\sin^{2}\left(\frac{\theta_{a}}{2}\right)+4l_{b}^{2}\sin^{2}\left(\frac{\theta_{b}}{2}\right)-8l_{a}l_{b}\cos\left(\frac{\theta_{a}}{2}\right)\cos\left(\frac{\theta_{b}}{2}\right)-8l_{a}^{}l_{b}^{}\cos\left(\theta_{3d}\right)},$$

Note also that, since the 2D projections of the tetrahedra are squares, one may compute the projected lenghts of the projected squares from their diagonals using Pythagoras theorem, which are given by:

(20) $$s_{a}=\frac{1}{\sqrt{2}}d_{a}=\frac{1}{\sqrt{2}}l_{a}\sin\left(\frac{\theta_{a}}{2}\right),$$

(21) $$s_{b}=\frac{1}{\sqrt{2}}d_{b}=\frac{1}{\sqrt{2}}l_{b}\sin\left(\frac{\theta_{b}}{2}\right),$$

whilst the angles between these squares are given by (i.e., the angle between the sides of the squares):

(22) $$\theta_{s}=\theta_{2d}-90^{o},$$

where $\theta_{2d}$ is the angle between the diagonals of the squares (corresponding to the angle between the tetrahedral ‘Set 1′ edges lying in the (001) plane), given by:

(23) $$\theta_{2d}=\cos^{-1}\left(\frac{4l_{a}^{}l_{b}^{}\cos\left(\theta_{3d}\right)+h_{a}^{}h_{b}^{}}{d_{a}^{}d_{b}^{}}\right)=\cos^{-1}\left(\frac{\cos\left(\frac{\theta_{a}}{2}\right)\cos\left(\frac{\theta_{b}}{2}\right)+\cos\left(\theta_{3d}\right)}{\sin\left(\frac{\theta_{a}}{2}\right)\sin\left(\frac{\theta_{b}}{2}\right)}\right).$$

Note that these geometric relationships are only applicable if the system is made from tetrahedra which are tetragonal disphenoids that are oriented in space and stacked as described here. Note also that Equations (5) and (19) for the cell parameters do not involve any reference to the torsion angles.

#### 2.2.2. Quantitative Comparison and Verification of Derivation

Table 1 gives the angles measured in degrees for the obtained structures. Table 2 compares the values of the various parameters associated with the mathematical model derived above computed using the experimentally derived structures and simulations.

First and foremost, this comparison confirms in a quantitative manner that the simulation protocol used is appropriate for simulating the crystal structure of BAsO_4_ at ambient pressure. In fact, the DFT simulations adequality reproduce the structure of boron arsenate and all the observations and measurements made on the experimentally obtained structures related to the shape, size and orientation of tetrahedra were replicated by the simulations.

Moreover, the mathematical representation of BAsO_4_ derived above was found to be fully applicable to the experimental and simulated structuces with the predicted values of the cell parareters as estimated through the mathematical model agreeing perfectly with the ones measured directly. In fact, as shown in Table 2, the predition holds perfectly (100% agreement) for the data obtained experimentally, i.e., *c_eqn_* the value of *c* predicted by Equation (6) is exacly identical to *c_act_*, the value of *c* actually measured and the values of *a* and *b* as computed through Equation (19) are equal to those actually measured directly in the experiment and simuations. Moreover, the rule proposed by McNelis and Blandino [60] was also found to be fully applicable to the simulated crystal structure, as shown in Table 1.

**Table 1:** The O-X-O (X = As, B) angles, in degrees, as measured from experimentally / DFT obtained structures. The protocol used for the simulations is outlined in Appendix A and the O-X-O angles are defined in Figure 2. Note that for each tetrahedron, the sum of the six O-X-O angles is approximately 657° in accordance with McNelis and Blandino [60].

		Schulze [2]	Haines [3]	Simulated		Schulze [2]	Haines [3]	Simulated
Set 1	O1-A1-O3, O5-A2-O7	111.4	114.1	115.2	O3-B1-O1, O7-B2-O5	113.2	111.0	113.9
	O2-A1-O4, O6-A2-O8	111.4	114.1	115.2	O8-B1-O6, O4-B2-O2	113.2	111.0	113.9
Set 2	O1-A1-O2, O5-A2-O6	108.5	107.2	106.7	O3-B1-O8, O7-B2-O4	107.6	108.7	107.3
	O2-A1-O3, O6-A2-O7	108.5	107.2	106.7	O8-B1-O1, O4-B2-O5	107.6	108.7	107.3
	O3-A1-O4, O7-A2-O8	108.5	107.2	106.7	O1-B1-O6, O5-B2-O2	107.6	108.7	107.3
	O4-A1-O1, O8-A2-O5	108.5	107.2	106.7	O6-B1-O3, O2-B2-O7	107.6	108.7	107.3
	**Sum (degrees):**	**656.9**	**657.0**	**657.1**	**Sum (degrees):**	**657.0**	**656.8**	**657.0**

**Table 2:** The various experimentally/DFT obtained parameters and structural features. Parameters defined in text.

		Schulze [2]	Haines [3]	Simulated
Bond lengths, as measured	*l_a_* (Å)	1.544	1.511	1.695
	*l_b_* (Å)	1.436	1.481	1.479
Bond angles, as measured	*θ_a_* (deg.)	111.4	114.1	115.2
	*θ_b_* (deg.)	113.2	111.0	113.9
	*θ_3d_* (deg.)	132.6	132.0	128.6
Tetrahedral heights	*h_a_* (Å)	1.740	1.644	1.816
	*h_b_* (Å)	1.580	1.679	1.614
Tetrahedral Set 1 edges	*d_a_* (Å)	2.550	2.535	2.862
/Square Diagonals	*d_b_* (Å)	2.399	2.441	2.479
	*θ*_2_*_d_* (deg.)	122.1	121.4	118.0
Projected Squares	*s_a_* (Å)	1.803	1.793	2.024
	*s_b_* (Å)	1.696	1.726	1.753
	*θ_s_* (deg.)	32.1	31.4	28.0
Unit Cell parameters *	*a*, *b* (Å)	4.332 (4.332)	4.340 (4.340)	4.582 (4.582)
	*c* (Å)	6.640 (6.640)	6.647 (6.647)	6.859 (6.859)
	*α*,*β*,*γ* (deg.)	90	90	90

* Note that the cell parameters as estimated by the metathetical model (*a_eqn_*, *b_eqn_* and *c_eqn_*), shown in parenthesis, agree perfectly with *a_act_*, *b_act_* and *c_act_*, the ones measured directly.

## 3. Boron Arsenate at Non-Ambient Pressures

### 3.1. General Considerations

**Figure 5:**
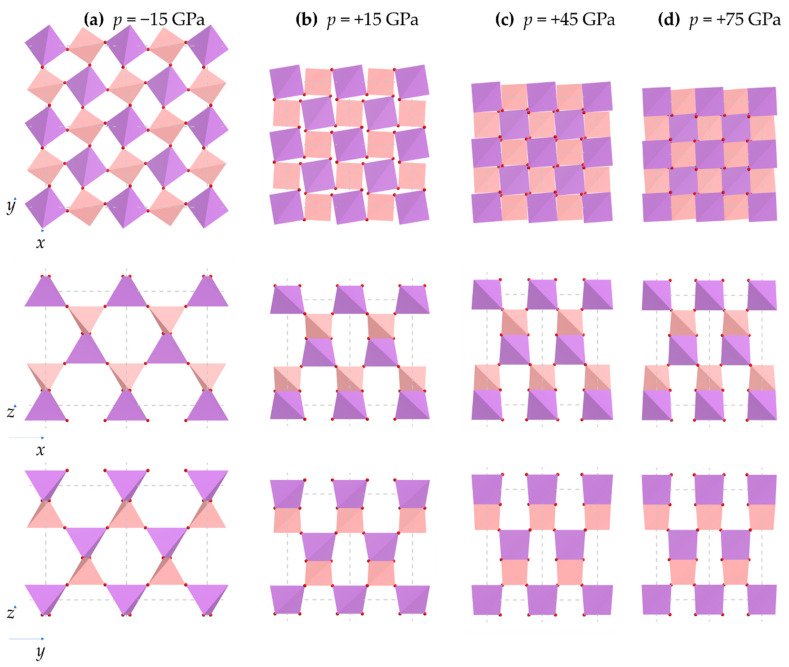
Images of the systems at various extents of applied pressure (**a**) −15 GPa, (**b**) +15 GPa, (**c**) +45 GPa, and (**d**) +75 GPa, with changes in the crystal structure being very evident. These changes are also very evident by the differences in reflections/peak positions in the PXRD patterns, see Appendix B.

**Figure 6:**
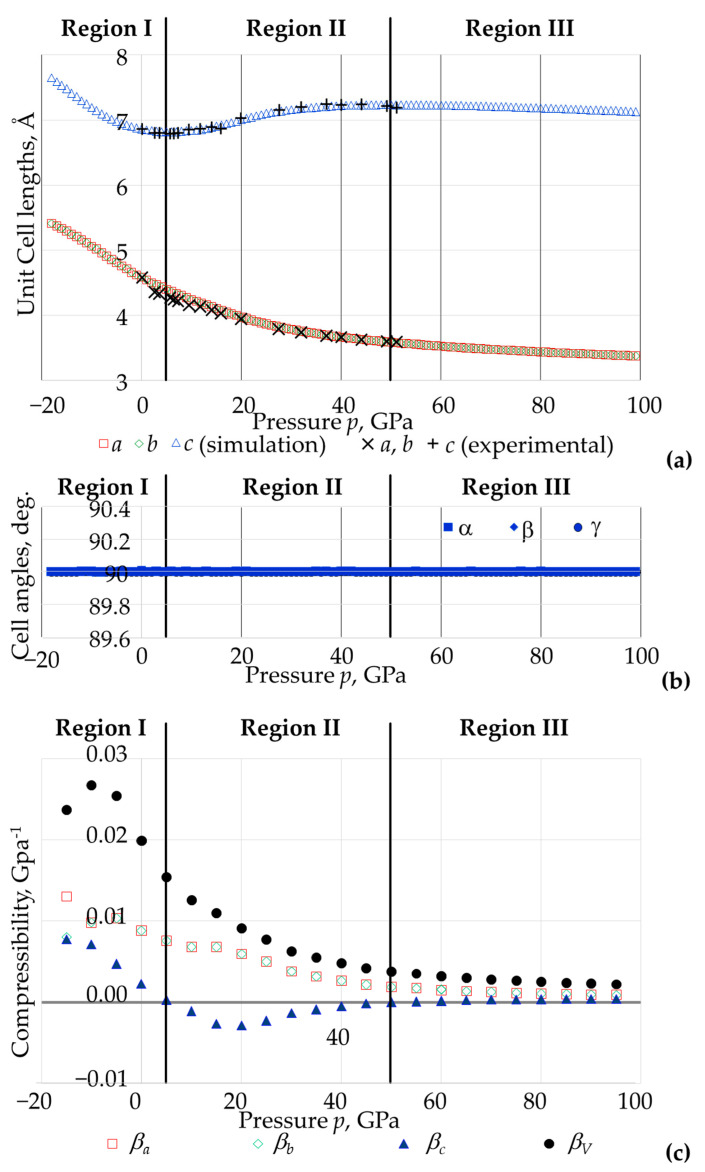
(**a**) The lattice constants *a*, *b*, *c* as simulated compared with the experimental data (shown as black crosses) as obtained by Haines et al. [3]. Note the excellent agreement between the simulated data and the available experimental data. (**b**) A confirmation that the lattice cell angles remain 90° throughout the entire pressure range considered means that lattice vectors *a*, *b* and *c* remain aligned with the *x*, *y* and *z* directions respectively. (**c**) The linear and volume compressibility values as computed from the simulated data. Note that Region II corresponds to NLC in the *c* direction.

Images showing the projection of the tetrahedra in the (001), (010) and (100) planes at pressures of −15 GPa to +75 GPa in intervals of 30 GPa are shown in Figure 5. Note that given that experimental data at non-ambient pressures over a wide pressures range is not readily available, this work relies on DFT simulations to obtain the crystal structure of BAsO_4_ over a wide pressure range, comparing whenever possible to existing experimental data (see Figure 6a).

A visual comparison of these systems indicates that an increase in pressure results in very prominent shrinkage in the *a* and *b* unit cell dimensions together with a visible re-orientation of the tetrahedra, where each tetrahedron rotates about an axis which is parallel to the [001] direction and corresponds to the aforementioned perpendicular bisector of the Set 1 edges of these tetrahedra, as can be explained graphically in Figure 5. One may also see some less prominent changes in the *c* dimensions.

These images further suggest that the tetrahedral rotations and re-orientations are such that the Set 1 edges appear to remain parallel to the (001) plane. On much closer inspection, one may also note deformations of the tetrahedra themselves. These deformations are, somewhat unfortunately, overshadowed by the more prominent aforementioned tetrahedral rotations making it imperative to resort to a quantitative approach to analyse the effect of pressure on the BAsO_4_.

### 3.2. The Effect of Pressure on the Overall Size and Shape of the Unit Cell in Terms of Lattice Constants and Compressibility

Plots of the cell parameters as a function of the applied pressure are shown in Figure 6a,b, whilst a plot of the compressibility as a function of the applied pressure is shown in Figure 6c. Note that the plot of the cell angles with *p* confirms that the unit cell remains cuboidal at all pressures, i.e., at all pressures, the compressibility in the *x*, *y* and *z* directions is always equal to the compressibility in the directions of the crystallographic axes *a*, *b*, *c*. Thus to simplify the discussion, the compressibility will be reported and discussed in terms of the crystallographic axes in analogy with earlier work by Haines et al. [3].

A first observation that should be made is that, as shown in Figure 6, the data related to unit cell lengths generated through simulations fits and compliments perfectly the existent data that were obtained experimentally by Haines et al. [3]. In fact, the trends in the variation of the cell parameters shown in Figure 6a, including the rather complex relationship between the cell parameter *c* and pressure, are practically identical. This observation is of paramount importance as these lattice parameters can be considered as the ‘raw data’ for this work, from which, the linear and volumetric compressibility properties are calculated, see Figure 6c, as explained in Appendix A. This suggests that computed compressibility values can be considered as realistic and validated.

More importantly, because this present work looks at a much wider range of pressures, the trends in the variation of the various paraments with pressure are now more clear, evident and identifiable. More specifically, the plots of the cell lengths with pressure suggest that whilst there is a simple trend in the behaviour of the cell parameters *a* and *b* with pressure *p*, which merely decease monotonically with a positive increase in the applied hydrostatic pressure (and remain equal at all pressures), the relationship between the *c* parameter with *p* is much more complex.

In fact, the *c* vs. *p* and *β_c_* vs. *p* plots (see Figure 6a,c), suggest that one may describe the compressibility properties in the *c* direction (i.e., the direction where NLC is exhibited) in terms of three rather distinct regions:

Region I: At low (below *c*. 5 GPa) and negative pressures, where the slope of the *c-p* plot is negative and steep at high negative pressures meaning that the compressibility is predicted to be positive and large;

Region II: At intermediate positive pressures (from *c.* 6 GPa to *c.* 50 GPa) where the slope of the *c*–*p* plot is positive and increasingly less steep meaning that the compressibility is negative (negative linear compressibility, NLC) with the highest magnitudes in the region of 15–25 GPa;

Region III: At extreme high positive pressure (from *c*. 51 GPa to *c*. 100 GPa) where the slope of the *c*–*p* plot is positive but less steep when compared to Region I, meaning where the compressibility is positive but of much lower magnitude.

Moreover, instances where *β_c_* = 0 in Figure 6c correspond to a scenario where the material can be deemed as being is incompressible in the *c* direction whilst still undergoing considerable compression in the orthogonal *a* and *b* directions. These instances where *β_c_* = 0 correspond to the transitions between Region I and II and between Region II and Region III. Note that in Region III, *β_c_* has a very low magnitude and the volumetric compressibility *β_V_* mainly arises from *β_a_* and *β_b_*.

Putting these findings in context of earlier work, one may note that Region II was very clearly identifiable in accordance to the report by Haines et al. [3] which studied BAsO_4_ from ambient pressures till c. 50 GPa (i.e., focused primarily on Region II). With the additional data reported here, which puts existing data into context, it can be clearly deduced that this NLC region lies between two other regions (which include the extreme positive and negative pressure conditions) where the compressibility in the *c*-direction is positive (and overwhelmingly so at negative pressures).

Moreover, as shown in Figure 6c, it is important to highlight that the volumetric compressibility is always positive, tending to zero at highly elevated pressures. This confirms that the bulk modulus *K*, which is the reciprocal of the volume compressibility, is always positive as required by classical thermodynamic considerations [53].

### 3.3. The Effect of Pressure on the Shape and Size of The BO*_4_* and AsO*_4_* Tetrahedra

**Figure 7:**
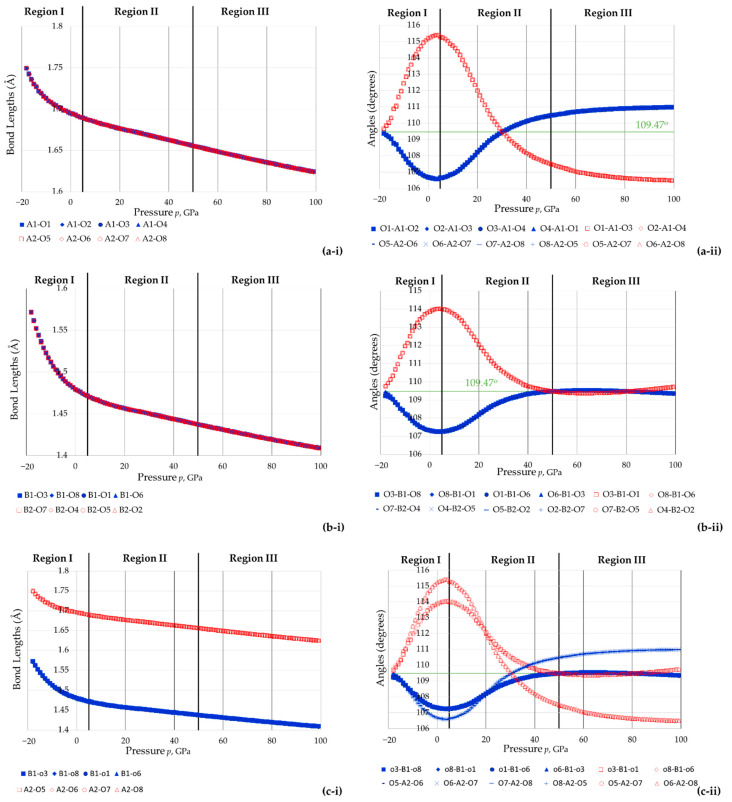
Data relating to (**a**) the two AsO_4_ tetrahedra, (**b**) the two BO_4_ tetrahedra, and (**c**) comparison of AsO_4_ and BO_4_ tetrahedra showing the variation in pressure of (**i**) bond lengths and (**ii**) bond angles.

Plots of the bond lengths and intra-tetrahedral bond angles for different tetrahedra within the unit cell of the system as estimated through the simulations are reported in Figure 7 below, where (a) shows the data relating to the two AsO_4_ tetrahedra within the unit cell, (b) shows the data relating to the two BO_4_ tetrahedra within the unit cell and (c) compares an AsO_4_ tetrahedron with a BO_4_ tetrahedron. First and foremost, the plots in (a) show that the two AsO_4_ tetrahedra remain perfectly identical in shape and size throughout the range of pressure considered, as do the two BO_4_ tetrahedra (see plots in (b)).

**Figure 8:**
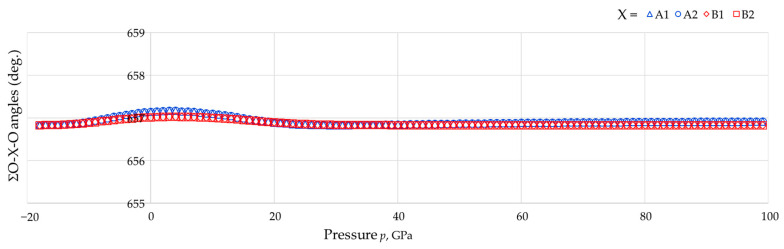
A plot of the sum of the six O-As-O intra-tetrahedral angles or six O-B-O angles pertaining to a single tetrahedron where it is shown that, subject to very minor discrepancies, the sum of angles always sums up to a constant value of *c*. 657°.

Moreover, throughout the pressure range considered:

(i) The eight As-O bonds remain of equal length, as do the eight B-O bonds;
(ii) The bond angles were found to retain their equality characteristics and could be divided into two types, Set 1 (shown in red in the graphs) and Set 2 (shown in blue in the graphs);
(iii) For each tetrahedron, the sum of the six angles O-As-O or O-B-O remain at approximately 657° throughout the whole range of pressures studied (see Figure 8, average: 656.89°, standard deviation: 0.08°, difference between maximum and minimum value: 0.34°).

This latter finding suggests, for the first time, that the rule proposed by McNelis and Blandino [60] is not just observed at atmospheric pressure but even at the extreme pressures such as the ones considered here. This aspect merits further investigation to assess the applicability of this rule at elevated pressures by other compounds.

More importantly for the purpose of this work, when findings (i) and (ii) are considered together, it is evident that the tetrahedra remain ‘tetragonal disphenoids’ (i.e., where the four faces are equal isosceles triangles). This hypothesis was further verified by plotting the edge lengths of the tetrahedra and the face angles. It is important to note that the finding that the tetrahedra retain their ‘tetragonal disphenoid’ shape throughout the pressure range considered means that all the geometric characteristics associated with having this shape are retained, including the property that the height of the tetrahedra are given by Equations (4) and (5) above.

Looking more closely on the variation of the As-O and B-O bond lengths and the O-As-O and O-B-O bond angles as reported in Figure 7 one may observe that although the general trends on how the AsO_4_ and BO_4_ tetrahedra behave are somewhat similar, they differ from each other in a number of aspects. For example, an analysis of the angles reveals that the smaller BO_4_ tetrahedra seems to become more regular (*T_d_* symmetry) as the pressure increases with quasi perfect *T_d_* symmetry at both very high positive pressures and negative pressures. In fact, at pressures higher than 50 GPa, the six O-B-O angles remain with the narrow range of 109.34° to 109.73° (i.e., very close to the value of 109.47°) of the regular tetrahedron with T_4_ symmetry (ideal behaviour being observed at *p* = 50 GPa, *p* = 81 GPa). In contrast, the larger AsO_4_ tetrahedra seem to retain their *T*_2*d*_ symmetry, and although they are simulated as having higher *T_d_* symmetry at *c*. 30 GPa, at higher pressures this symmetry reverts back to *T*_2*d*_, with the Set 1 bond angles now being smaller than the ideal tetrahedral angle of 109.47° whilst the Set 2 bond angles have higher values. On the other hand, it seems that the B-O bonds are comparably amenable to being compressed (positive pressure) when compared to the As-O bond. In fact, the magnitude of decrease in length of the As-O bond when pressure was changed from *p* = 0 GPa to *p* = 100 GPa was 0.0713 Å (4.21% shrinkage), whilst the B-O bond length decreased by a comparable amount of 0.0709 Å (4.79% shrinkage). On the other hand, the B-O bond was much more amenable to being stretched upon application of negative pressure from *p* = 0 GPa to *p* = −15 GPa with the B-O bond length increasing by 0.0644 Å (4.35% increase), which is almost double the increase experienced by the As-O bond 0.0356 Å (2.10% increase). These differences may be explained through a chemo-mechanical explanation as, in the smaller BO_4_ tetrahedra, much shorter lengths and angles less than 109.88° would mean that the oxygen atoms, which are electronegative, would be too close and hence repel each other. Such steric effects are less pertinent to the larger AsO_4_ tetrahedra, with the result that the Set 1 angles can keep shrinking past the 109.47° angle. In a similar way, the more pronounced extension of the B-O bond length on application of a negative pressure can be seen as a manner to relieve the O-O repulsions which would occur in such small BO_4_ tetrahedra.

**Figure 9:**
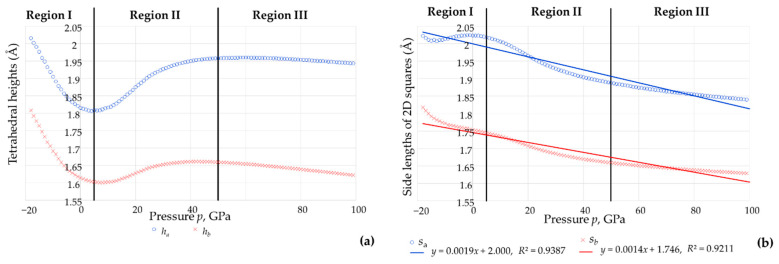
The relationships with pressure *p* of (**a**) the tetrahedral heights for the AsO_4_ and BO_4_, *h_a_* and *h_b_* and (**b**) the length of the projected squares in the (001) plane, *s_a_* and *s_b_*.

Having established that the tetrahedra retain the shape of a tetragonal disphenoid, one may make use of the expressions derived above to evaluate how the height and ‘square’ projection of the tetrahedra change with pressure. Plots of the height of the tetrahedra *h_a_* and *h_b_* with pressure are shown in Figure 9a whilst plots of the lengths of the sides of the squares *s_a_* and *s_b_* with pressure are shown in Figure 9b. These images show that the variation of these tetrahedral parameters is more complex when compared to that of the bond length, with the tetrahedral heights generally following the trends in the changes in the cell parameter *c* and the intra-tetrahedral bond angles with pressure. In particular, both *h_a_* and *h_b_* are at a minimum in the proximity of 5 GPa, i.e., the pressure where the compressibility in the *c* direction changes sign from positive in Region I to negative in Region II. For both tetrahedra, this minimum height at the transition from Region I at Region II is so pronounced that the height is at the absolute minimum of the entire −18 GPa to 100 GPa range studied. At pressures higher than 5 GPa, the tetrahedral heights increase in magnitude till reaching a maximum, and then start decreasing slightly again. Note that these maximum turning points are much less pronounced when compared to the minimum turning point at *c*. 5 GPa and do not coincide exactly with the Region II-Region III transition, but one occurs at a lower pressure and one at a higher pressure with maximum turning point for the AsO_4_ tetrahedra occurring at *c*. 43 GPa whilst that for the BO4 tetrahedra occurs at *c*. 60 GPa.

The relationship between the side lengths of the 2D projected squares *s_a_* and *s_b_* with pressure is equally complex. In fact, although one can clearly identify a number of turning points, including one which occurs at negative pressures (Region I) and one which occurs at around the transition from Region I to Region II, the variation is such that, to a very rough first approximation, *s_a_* and *s_b_* vary in a quasi linear manner with pressure (*R*^2^ > 0.9). This tallies with the visual observation that the projected squares in the (001) plane can be seen to get smaller with an increase in pressure.

### 3.4. The Effect of Pressure on the Orientation of the BO*_4_* and AsO*_4_* Tetrahedra

**Figure 10:**
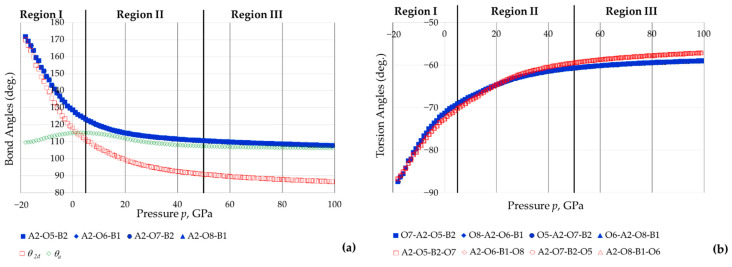
(**a**) The variation with pressure *p* of the inter-tetrahedral B-O-As angles (collectively known as *θ*_3_*_d_* and their 2D projection (*θ*_3_*_d_*) compared to a typical intra-tetrahedral bond-angle change; (**b**) the variation with pressure *p* of the torsion angles.

**Figure 11:**
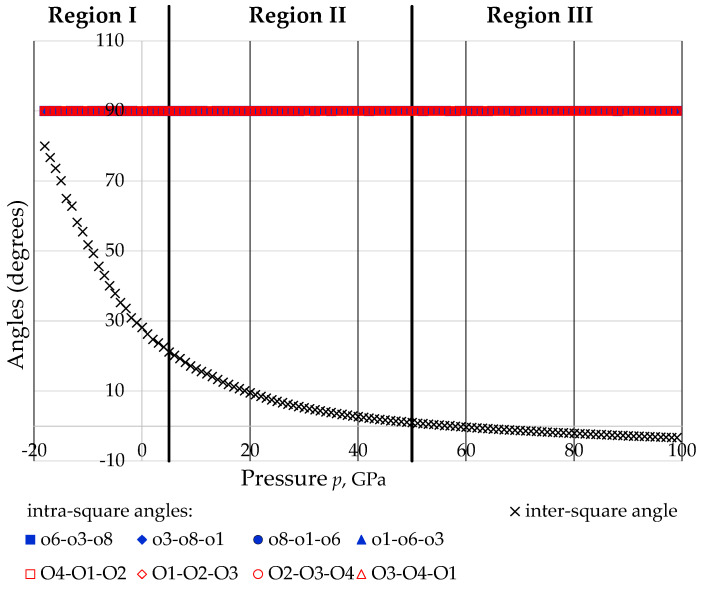
The variation with pressure *p* of the intra and inter-square angles with the intra-square angles remaining practically constant at 90° throughout the whole range of applied pressures, whilst the inter-square angle tending to 90° at high negative pressures (‘fully open’ conformation) and to 0° (‘fully closed’ conformation) at extreme positive pressures. Note that at *p* = 10 GPa, the inter-square angle had already decreased to just *c.* 16° and at *p > c.* 58 GPa, the inter-square angle is less than 0°. This may be permitted since the tetrahedra which project as squares occupy different ‘depth’ down the *c*-direction, i.e., do not physically overlap.

Plots of the As-O-B bond and torsion angles with pressure are shown in Figure 10a, together with the 2D projected angle between the diagonals and the corresponding angle between the squares. Figure 11 shows plots with angles corresponding to the 2D squares (intra-square ad inter-square). These plots suggest that:

(i) All the As-O-B inter-tetrahedral bond angles remain equal to each other throughout the range of pressures considered, and that there is a monotonic decrease in these angles with an increase in pressure;
(ii) The As-O-B angles change considerably with pressure, much more than any other parameter in the system (see comparison with a representative O-As-O *θ_a_* angle in Figure 10a), indicating that there are pronounced tetrahedral rotations though ‘tilting’ in the manner illustrated in Figure 3;
(iii) The torsion angles also vary extensively with pressure (see Figure 10b), indicating that there is considerable tetrahedral ‘twisting’ in the manner illustrated in Figure 3;
(iv) There is extensive change in the angles between the 2D projected squares (the ‘inter-square’ angle) with practically no change in the 90° intra-square angles (see Figure 11).

An interpretation of these changes needs to be made keeping in the mind what is clearly observable in Figure 5, i.e., the very evident rotations/tilting/twisting of the tetrahedra (which have already been proved to remain tetragonal disphenoids) seem to be such that apart from deforming, the tetragonal disphenoids rotate around a local axis which is parallel to the *c*-direction, i.e., retain their Set 1 edges parallel to the (001) plane. Such net rotations, which preserve the tetragonal disphenoidal characteristics of tetrahedra and the $I\bar{4}$ overall symmetry of the crystal, cannot be accomplished through either changes in the As-O-B bond angles alone, or changes in the torsion angles alone, but require a synergistic change of these parameters simultaneously. In other words, although the torsion angles do not feature in the expressions for the cell parameters derived above, the torsion angles must change with pressure to relieve the internal stresses within the system such that the overall deformation is the one which preserves the symmetry.

**Figure 12:**
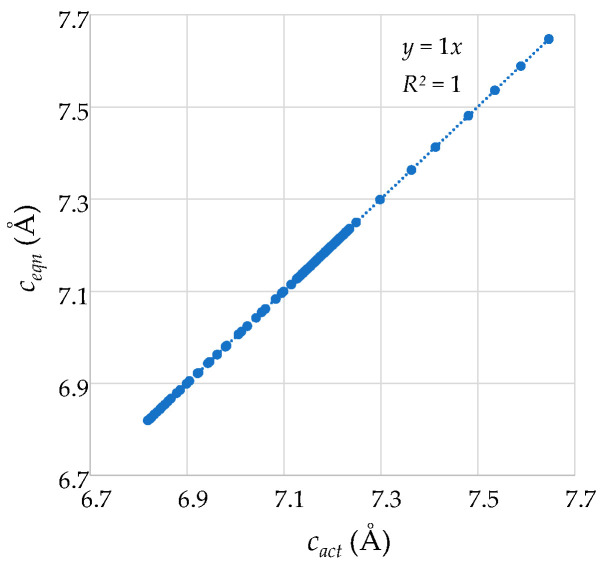
A graphical confirmation that the directly measured unit cell length in the *c* direction (*c_act_*) tallies exactly with the value computed from adding the heights of the tetrahedra (*c_eqn_*).

Given that it has already been verified that the tetrahedra retain the shape of tetragonal disphenoids over the whole pressure range considered, the hypothesis that the tetrahedra are indeed aligned in this manner (i.e., where the Set 1 edges are parallel to the (001) plane and the $I\bar{4}$ symmetry characteristics are preserved) can be verified by confirming that the perpendicular bisector of the Set 1 edges is parallel to the *c* direction at all pressures. Should this be the case, given the connectivity, the unit cell length in the *c* direction would equate to maximum distance attainable from the combined height *h* of the four tetrahedra within the unit cell, i.e., their sum. This was indeed verified through the measurements obtained from the DFT simulations which confirmed that Equation (5) above remained applicable at all pressures, i.e., the height of the unit cell in the *c* direction always equates to the summed height of the four tetrahedra measured as the length of perpendicular bisector of the Set 1 edges. This verification, shown in Figure 12 through a plot of ‘*c_eqn_* = 2*h_a_* + 2*h_b_*’ vs. ‘*c_act_’* which fits to *y = x* perfectly with *R^2^* = 1, also explains why the negative compressibility characteristics in the *c* direction are so much relatable to the manner how the height of the tetrahedra varies with pressure explained above.

Another very interesting result which stems out from the results of the DFT simulation as reported through the images in Figure 5 and the plots in Figure 10a and Figure 11 is the extent to which the projected 2D squares in the (001) plane, with the characteristic ‘rotating squares’ motif, rotate relative to each other with changes in pressure and the extent with which *θ*_2*d*_, the 2D projected angle between the diagonals, changes with pressure. In fact, the simulations and calculations suggest that this system undergoes changes in *θ*_2*d*_ from *c.* 170° at −18 GPa (180° would have corresponded to the fully open ‘rotating squares’ motif, or 90° in Figure 11) to *c.* 87° at 100 GPa, i.e., slightly more closed than 90° that one normally considers for the fully closed ‘rotating squares’, something which is possible since the AsO_4_ and BO_4_ tetrahedra, the units which are actually physically rotating, occupy distinct physical positions down the *c* direction. This range of values, which roughly corresponds to the full range of values applicable to the ‘rotating squares’ model, emphasises the robustness of this mechanistic model and proves its applicability to boron arsenate under hydrostatic pressure loading. Here it should be noted that the ‘rotating squares’ mechanistic model is one which can take considerable compression and expansion (it can increase or shrink in size by up to 100%) thus explaining why this material is so extensively compressible in the *a* and *b* directions.

## 4. Discussion

This work, through the extensive DFT-based simulations and through the use of geometry-mechanism-based models, looks into the behaviour of BAsO_4_ over a very wide range of pressures, which include extreme pressures through 100 GPa and negative pressures. Through these simulations, models and expressions, it was possible to investigate and report important information on how this crystalline material behaves under pressure, with an emphasis on nanoscale atomic-level deformations that occur.

An important finding was the confirmation that the compressibility characteristics in the *c* dimension are completely attributable to changes in shape and size of the tetrahedra, and not changes in their relative orientation. Having established this fact and having derived an expression to mathematically describe how the unit cell parameter *c* may be computed in terms of the geometric parameters quantifying the bond lengths and bond angles (Equation (5)), one may make use of this equation to delve further into what gives rise to NLC in the *c* direction in Region II, or otherwise in Regions I and III, and, for the first time, quantify to what extent.

It is evident from Equation (5) that from the many geometric properties which characterise BAsO_4_, the cell parameter *c* is dependent on the bond lengths *l_a_* and *l_b_* and the bond angles *θ_a_* and *θ_b_*. Thus, the compressibility will depend upon:

(1) The manner how these four parameters change with pressure, which is directly relatable to the slope of the parameter with pressure;
(2) The ‘weighting’ that needs to be given to this change in parameter to the overall deformation.

Since the linear compressibility at constant temperature *T* conditions is defined as:

(24) $$\beta_{c}=-\frac{1}{c}{\left(\frac{\partial c}{\partial p}\right)}_{T},$$

this information, mathematically, can be obtained from the full derivate of *c_eqn_* in Equation (5), given by:

(25) $$dc_{eqn}=\frac{\partial c_{eqn}}{\partial l_{a}}dl_{a}+\frac{\partial c_{eqn}}{\partial l_{b}}dl_{b}+\frac{\partial c_{eqn}}{\partial \theta_{a}}d\theta_{a}+\frac{\partial c_{eqn}}{\partial \theta_{b}}d\theta_{b},$$

which, if the changes in the parameters are due to a change in pressure *dp*, may be re-written as:

(26) $$\frac{dc_{eqn}}{dp}=\frac{\partial c_{eqn}}{\partial l_{a}}\frac{dl_{a}}{dp}+\frac{\partial c_{eqn}}{\partial \theta_{a}}\frac{d\theta_{a}}{dp}+\frac{\partial c_{eqn}}{\partial l_{b}}\frac{dl_{b}}{dp}+\frac{\partial c_{eqn}}{\partial \theta_{b}}\frac{d\theta_{b}}{dp},$$

i.e., from Equations (5) and (24)–(26):

(27) $$\beta_{c}=\left[-\frac{4}{c}\cos\left(\frac{\theta_{a}}{2}\right)\right]\frac{dl_{a}}{dp}+\left[-\frac{4}{c}\cos\left(\frac{\theta_{b}}{2}\right)\right]\frac{dl_{b}}{dp}+\left[\frac{4l_{a}}{c}\sin\left(\frac{\theta_{a}}{2}\right)\right]\frac{d\theta_{a}}{dp}+\left[\frac{4l_{b}}{c}\sin\left(\frac{\theta_{b}}{2}\right)\right]\frac{d\theta_{b}}{dp},$$

where the derivatives equate to the manner how the four parameters change with pressure, which may be estimated by the slope of the parameter with pressure, and the terms in the square brackets are the respective ‘weighting’ that needs to be given to these changes.

First and foremost, recognising that since cell parameter *c*, bond lengths *l_a_* and *l_b_* are obviously always positive and the results of the simulations suggest that the angle values for *θ_a_* and *θ_b_* range from *c.* 106.5° to 115.5° (i.e., the sine and cosine of half these angles is always positive), one may note that the first two terms in the square brackets, relating to changes in bond lengths, are negative and the last two terms, relating to changes in bond angles, are positive. Thus, for NLC in the *c* direction (negative *β_c_*), one would need:

(i) Positive slopes in the variation with the bond length with pressure, which is not the case, since these slopes are always negative for BAsO_4_ (and most other known materials), signifying that the bond lengths are shrinking with an increase in pressure, and/or
(ii) Negative slopes in the variation of the specific angular values *θ_a_* and *θ_b_* with pressure, which is the case for BAsO_4_.

Analysing this in a more quantitative manner requires knowledge of the slopes and the manner how these vary with pressure, apart from knowledge of the actual parameters. For this specific study, the slopes were estimated at *p* = −15 GPa, −5 GPa, +5 GPa, …, +95 GPa, i.e., at intervals of 10 GPa, through a linear regression of the five data points within the narrow region of *p* ± 2 GPa, whilst the actual values of the geometric parameters at a pressure *p* were taken as the average of the appropriate measurements at that pressure (e.g., *θ_a_* was taken as the average of the four bond angles O1-A1-O3, O2-A1-O4, O5-A2-O7 and O6-A2-O8).

**Figure 13:**
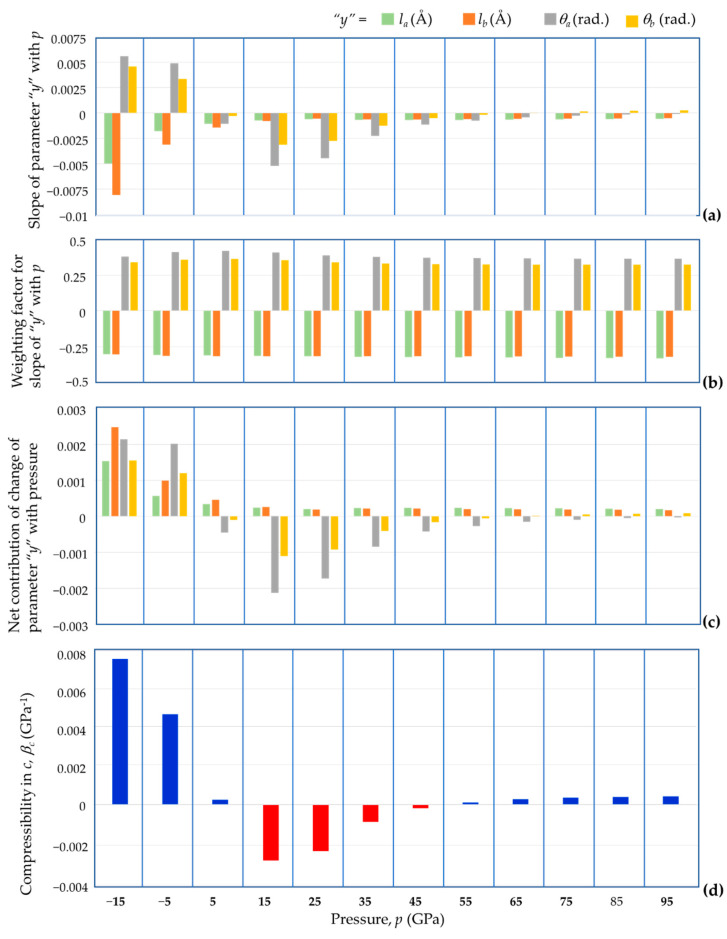
A graphical report of the different components; (**a**) slope of parameter *y*, (**b**) weighting factor of *y*, and (**c**) net contribution of change in *y*, which contribute to (**d**) the compressibility in the *c* direction (with negative compressibility shown in red in (**d**)).

This quantitative finding from this analysis is presented graphically though the bar chart in Figure 13, with Figure 13c indicating the sign and magnitude of the four contributions to the compressibility. This plot is most informative in assessing what gives rise to NLC or otherwise (see Figure 13d), and suggests that in Region II, the main net contributor to NLC in the *c*-direction is the change in the O-As-O bond angles (which causes an increase in height in the AsO_4_ tetrahedra with increasing pressure) followed by O-B-O bond angles (which causes an increase in height in the BO_4_ tetrahedra with increasing pressure).

In contrast, the change in bond lengths always contributes to positive compressibility, something which they do for all three regions. It is interesting to note that in the negative pressure portion of Region I, where high positive compressibility is observed, all the four parameters contribute to positive compressibility. In Region III, where low positive compressibility values are measured, the changes in the O-As-O bond angles keep contributing to an increase in *c* through an increase in height of the AsO_4_ tetrahedron (this is not the case with the O-B-O angles), but the net shrinkage (positive contribution) resulting from the change in the other parameters cancels out and slightly outweighs the increase with the result that positive compressibility is observed.

**Figure 14:**
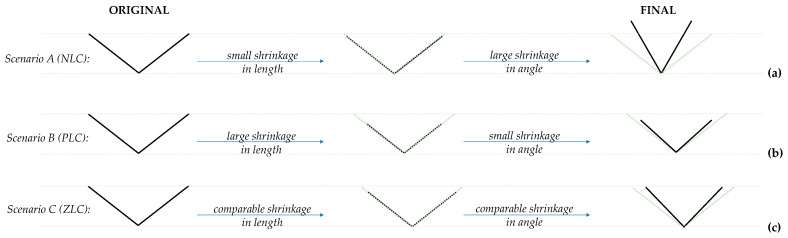
An illustration of how (**a**) negative linear compressibility (NLC), (**b**) positive linear compressibility (PLC) and (**c**) zero linear compressibility (ZLC) manifest in the vertical direction.

These results may be elucidated through the graphical illustration shown in Figure 14 which focuses on a basic V-shaped unit which corresponds to half of a tetrahedral unit. Such units, which can also be considered as half of the more well-known ‘standard wine-rack unit’ (hence the nomenclature of ‘demi wine-rack’ in Grima-Cornish et al. [4]), can deform in just two ways: either through a change in length (which in this case, due to symmetry consideration, they will deform equally), and/or, a change in angle. Assuming that under positive hydrostatic pressure, the deformation will be one having a shrinkage in the lengths and a shrinkage in the angle, the actual shape of the resulting unit could be either dominated by a change in angle or a change in length, where, as illustrated in Figure 14, one may have:

Scenario A: A deformation dominated by a change in angle would correspond to an elongation of the demi wine-rack height, i.e., an increase in a linear dimension when pressure is applied (negative linear compressibility, NLC, in the vertical direction);

Scenario B: A deformation dominated by a change in length would correspond to a shrinkage of the demi wine-rack height, i.e., a decrease in a linear dimension when pressure is applied (positive linear compressibility, PLC, in the vertical direction);

Scenario C: A deformation with comparable contribution from a change in length (corresponding to a shrinkage in height) and a change in angle (corresponding to an increase in height) which would cancel each other out resulting in no net change of the demi wine-rack height, i.e., no change in a linear dimension when pressure is applied (zero linear compressibility, ZLC, in the vertical direction).

Recognising that in this case, the V-shaped demi wine-rack units correspond to either two O-As bonds at angle O-As-O to each other, or, two O-B bonds at angle O-B-O to each other (half tetrahedra), from Figure 13, it is evident that Scenario A would clearly correspond to pressures of 15–45 GPa where change of angle dominates (Region II); Scenario C corresponds to around 5 GPa and somewhere in the region between 45–55 GPa, where the ‘positive’ and ‘negative’ contributions cancel each other out (transition between Region I and II and between Region II and III), and Scenario B corresponds to the extreme pressure regions of Region I and III. All this provides a formal verification of the cause for NLC (or otherwise) in terms of the ‘demi wine-rack’ mechanism.

**Figure 15:**
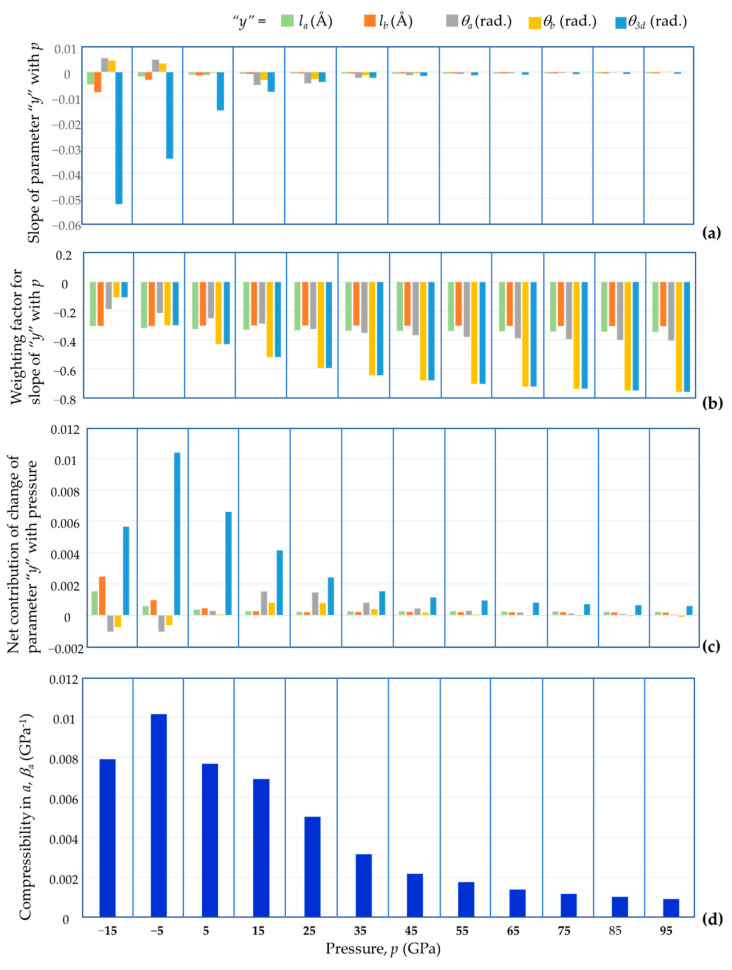
A graphical report of the different components (**a**) slope of parameter *y*, (**b**) weighting factor of *y*, and (**c**) net contribution of change in *y*, which contribute to (**d**) the compressibility in the *a* direction (similarly for the *b* direction).

A similar quantitative analysis, this time to look at the compressibility behaviour in the *a* direction, was also carried through a similar protocol (see Figure 15). In this case, since the dimensions of the unit cell in the *a* direction were found to be dependent on five geometric variables, i.e., *l_a_*, *l_b_*, *θ_a_*, *θ_b_* and *θ*_3_*_d_*, the analysis included a relative comparison of how and to what extent each of these five parameters contributes to the compressibility. As shown in Figure 15, which reports the results from this analysis, the change in *θ*_3_*_d_*, the inter-tetrahedral As-O-B angle, is the most important contributing factor to the compressibility in the *a* direction (and, by symmetry, the *b* direction). This finding agrees with the report made in the pioneering work by Haines et al. [3] who also identified, through similar simulations and experimental work, that tetrahedral rotations are the main type of deformation though which BAsO_4_ deforms when subjected to pressure changes.

Before concluding it is important to point out some of the main strengths and limitations of the present study. An important strength is its novelty: This work has, for the first time:

(1) Reported the predicted compressibility of BAsO_4_ over a much wider range of pressures compared to what was reported before, including very extreme pressures;
(2) Identified three rather distinct regions with different compressibility characteristics in *c* direction (one NLC region, two PLC regions);
(3) Analysed the structure of BAsO_4_ from a purely geometric perspective, in the process revealing various interesting features such as the tetrahedra are tetragonal disphenoids, a shape which is retained over a very wide range of pressure;
(4) Developed a new approach for examining compressibility by presenting a mathematical parametric model based on geometric and mechanistic considerations which permit the quantification of the relative contributions to the overall compressibility, both positive and negative, thus be able to unlock in a quantifiable manner what gives rise to NLC or otherwise;
(5) Proved in a quantitative manner than the proposed ‘demi wine-rack’ mechanisms involving tetrahedra deformations is indeed the mechanisms which leads to NLC, rather than the more conspicuous tetrahedra rotations;
(6) Verified that the rule proposed around two decades ago by McNelis and Blandino [60] concerning tetrahedral bond angles is not just applicable at ambient pressures but also at the extreme pressures such as the ones applied here.

An important limitation is that the work was based on simulations rather than experimental work. This means that no assessment has been made as to whether BAsO_4_ would remain stable at the extreme pressures considered here. However, it should be noted that experimental work by Haines et al. [3] has experimentally confirmed that this material can indeed sustain rather high pressures which exceed 50GPa. It is also comforting that despite these limitations, the simulated unit cell parameters, and manner these change with pressure (i.e., the compressibility), agree so well with the experimental findings as shown in Figure 6. Another obvious limitation is that, the part of the study which refers to subjecting the material to negative pressure represents a virtual scenario, since, in reality such ‘negative pressure’ conditions are not achievable experimentally. However, from a theoretical perspective, the study conducted here at such pressures is essential to have a complete picture of how this material behaves. In fact, an important strength of the present work is that it provides a much more complete picture of how BAsO_4_, a material which exhibits negative linear compressibility in the *c* direction, behaves under pressure, including a formal verification of the cause for NLC (or otherwise) in terms of the ‘demi wine-rack’ mechanism.

An even more important strength relates to the mathematical expressions derived here. It should be noted that although these expressions where derived specifically for BAsO_4_, it is likely that they would be applicable to other $I\bar{4}$ systems, as well as more symmetric systems and related materials, when subjected to a uniform hydrostatic pressure. Obviously, this hypothesis should first be tested since one would need to ascertain that the assumptions made in this work remain applicable. In particular, it should be noted that these expressions were based on the important assumption (confirmed in this case) that the tetrahedra remain tetragonal disphenoids. This was an empirical finding, and one should not expect that all tetrahedra would retain this property under asymmetric mechanical loading conditions, such as uniaxial loading.

Moreover, an important strength is this work provides the first confirmation that the rule proposed by McNelis and Blandino [60] is so robust that it seems to be fully applicable for materials at non-ambient pressures, where in this case it was tested at extreme pressures, both positive and negative. It would be useful if further similar studies are conducted to assess the applicability of this rule to other materials built from tetrahedral units, particularly in view of the fact that the applicability of this rule has important implications as it could facilitate similar work where the structure and properties are analysed through the use of mathematical geometry-based models.

## 5. Conclusions

This work looks in detail, through DFT simulations and mathematical models, into how boron arsenate behaves under moderate and very extreme pressure conditions. This material was studied in view of its anomalous negative linear compressibility (NLC) properties. A mathematical model which describes the crystal structure of BAsO_4_ in terms of geometric parameters for a wide range of pressure was derived. Through this model it was possible to unlock, in a quantifiable manner, what gives rise to NLC or otherwise and explain these phenomena in terms of geometric mechanistic models. The approach used, which was able to quantify the extent to which various geometric features which characterise BAsO_4_ contribute to NLC or otherwise, is rather unique and is expected to be transferable to other materials with similar features, characteristics or properties.

Another interesting outcome of this work is the verification that the rule proposed around two decades ago by McNelis and Blandino [60], which states that the sum of the tetrahedral bond angles can always be estimated at 657°, irrespective of chemical composition, is not just applicable at ambient pressures but also at elevated and extreme pressures such as the ones applied here. To our knowledge, this is the first time that this rule was tested at elevated and extreme pressures, and its applicability was confirmed.

It is hoped that this work will provide an impetus to researchers to not only experimentally verify the simulation and model results reported here, but also assess whether the models, predictions and explanations made here, including the applicability of McNelis and Blandino [60] rule at non-ambient conditions, are transferable to other materials and metamaterials.

## Figures and Tables

**Figure A1:**
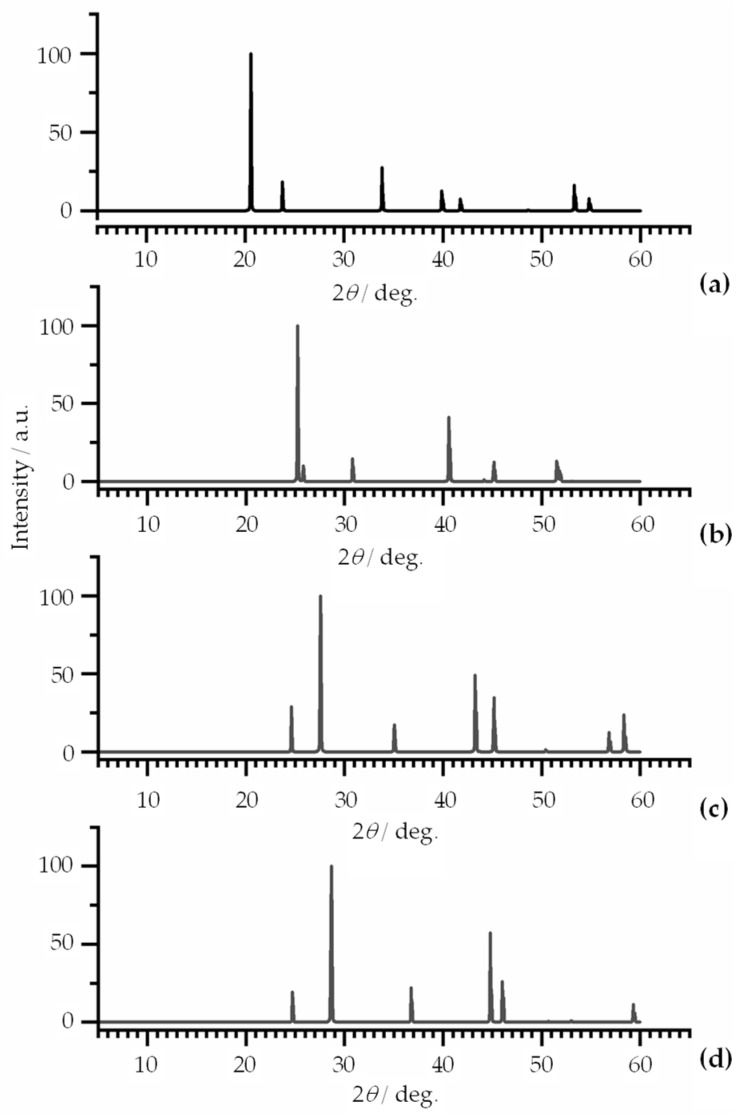
X-ray diffraction spectra (**a**–**d**) for the structure of BAsO_4_ corresponding to the systems at pressure *p* = −15GPa, 15GPa, 45GPa and 75GPa calculated using the Reflex module in Materials Studio. Note that changes in the crystal structure are very evident from the differences in reflections/peak positions.

## Data Availability

All data is available within this article.

## Appendix A. Calculation Method: DFT Simulation of BAsO_4_ at Ambient and On-Ambient Pressures

Simulations were carried out on a perfect single crystal using the CASTEP Density Function Theory (DFT) module within the Materials Studio modelling and simulation environment using the protocol outlined elsewhere [4]. Data obtained from these DFT simulations, together with available experimental data [1,2,3], was subsequently used to formulate the assumptions for the gemeortic model proposed in this paper.

In brief, the starting structure for the simulations was the 1 × 1 × 1 experimentally determined crystal structure as reported by Haines et al. [3] with the symmetry reduced from $I\bar{4}$ to P1. Full periodic boundary conditions were applied with no additional constraints being applied on the unit cell parameters. This unit cell contains twelve independent atoms (i.e., 3*n* − 3 = 33 degrees of freedom), namely two arsenic atoms (labelled A1 and A2), two boron atoms (labelled B1 and B2) and eight connecting oxygen atoms, labelled O1, O2, …, O8 as indicated in Figure 2.

Throughout the simulations, the standard convention as adopted by the Institute of Radio Engineers (IRE) for aligning the unit cell with the global coordinate system was used. By adopting this convention, the *c* crystal direction remains aligned parallel to the global *z*-axis, the *b* crystal direction remains aligned with the global *yz*-plane whilst the *a* crystal direction remains free. The application of this convention facilitates the measurements of the projections of the unit cell in the *x*, *y*, and *z* directions since the unit cell matrix **H**, even if the crystal lattice is not cuboidal, will always assume an upper-triangular form with the projections of the unit cell in the *x*, *y* and *z*-directions corresponding to the diagonal elements. This facilitates the reporting of on-axis dimensions and strains which is a key aspect of this study.

The energy expression was constructed using Generalised Gradient Approximations (GGA) simulations with the Perdew-Burke-Ernzerhof (PBE) exchange correlation functional [61,62]. As in previous works [4,5,6], the *k*-point separation was set to approximately 0.04 Å^−1^, which in the case of boron arsenate at ambient pressures corresponds to a Monkhorst-Pack Grid of 6 × 6 × 4 (actual spacing used was 0.037 Å^−1^). An energy cutoff value of 1200 eV was used. The overall charge was set to zero to indicate that the system is overall neutral. All atoms were assumed to be in their ground electronic state. The default Materials Studio ‘Fine’ setting was applied to the self-consistent field (SCF) tolerance whist the Fast Fourier Transform grid (FFT grid) was set to the default ‘precise’ setting. These settings correspond to an SCF tolerance value of 1 × 10^−6^ eV atom^−1^ with a maximum of 100 SCF cycles and an FFT grid of size 30 × 30 × 45.

Geometry optimisation was first carried out using the Broyden–Fletcher–Goldfarb–Shanno (BFGS) minimiser, making use of the line search method, where a pressure of 1 atm (i.e., 0.101GPa) was applied to replicate the structure of BAsO_4_ at ambient pressures. The system was considered as optimised if some rather stringent convergence criteria were satisfied. These included an energy cutoff per atom of 5 × 10^−6^ eV atom^−1^; a maximum force on each atom of 0.01 eV Å^−1^; a maximum atomic displacement of 0.0005 Å; and a maximum stress on the crystal of 0.001 GPa. These criteria were met prior to reaching a maximum of 1000 iterations.

Moreover, geometry optimisations using the protocol described above were carried for the system with the pressures being applied in an incremental manner starting from *p* = 0 GPa to extremely high pressure values of *p* = 100 GPa in increments of 1 GPa. A set of similar simulations were performed to simulate the rather hypothetical situation of ‘negative hydrostatic pressures’ to a maximum of –18 GPa.

The results obtained, apart from being compared to those reported by Haines et al. [3] whenever possible, were analysed in detail so as to identify the nanoscale changes in the crystal structure as a result of an applied pressure. Various geometric parameters were measured as a function of pressure which describe the crystal structure in terms of the bond lengths, bond angles and torsion angles, tetrahedral edge lengths and tetrahedra vertex angles.

Morover, the lattice constants *a*, *b*, *c* as a function of applied pressure were re-expressed in terms of linear compressibilies in the crystallographic *a*, *b* and *c* directions at a constant termperature *T* defined, respectively, as:

(A1) $$\beta_{a}=-\frac{1}{a}{\left(\frac{\partial a}{\partial p}\right)}_{T},$$

(A2) $$\beta_{b}=-\frac{1}{b}{\left(\frac{\partial b}{\partial p}\right)}_{T},$$

where the derivative terms in Equations (A1), (A2) and (24) are estimated as the slope of the cell parameter vs. *p*, estimated at a pressure *p* over five consecutive data points (i.e., over a pressure range of *p* ± 2 GPa) asuming a linear relatioship within the pressure range *p* ± 2 GPa.

Similary, the volume compressibility (and hence its reciprocal, the bulk modulus *K*) defined by:

(A3) $$\beta_{V}=\frac{1}{K}=-\frac{1}{V}{\left(\frac{\partial V}{\partial p}\right)}_{T}=\beta_{a}+\beta_{b}+\beta_{c}.$$

were estimated every 5 GPa, where *V* was taken as the unit cell volume at a pressure *p* and the derivative term was estimated as the slope at a pressure *p* of the unit cell volume vs. *p*, estimated over five consecutive data points asuming a linear relatioship within the pressure range *p* ± 2 GPa.
